# Impact of fecal sample preservation and handling techniques on the canine fecal microbiota profile

**DOI:** 10.1371/journal.pone.0292731

**Published:** 2024-01-29

**Authors:** Olivia Chiu, Diego E. Gomez, Dasiel Obrego, Kari Dunfield, Jennifer L. MacNicol, Brooklynn Liversidge, Adronie Verbrugghe

**Affiliations:** 1 Department of Clinical Studies, Ontario Veterinary College, University of Guelph, Guelph, ON, Canada; 2 School of Environmental Sciences, University of Guelph, Guelph, Ontario, Canada; Bayero University Kano, NIGERIA

## Abstract

Canine fecal microbiota profiling provides insight into host health and disease. Standardization of methods for fecal sample storage for microbiomics is currently inconclusive, however. This study investigated the effects of homogenization, the preservative RNA*later*, room temperature exposure duration, and short-term storage in the fridge prior to freezing on the canine fecal microbiota profile. Within 15 minutes after voiding, samples were left non-homogenized or homogenized and aliquoted, then kept at room temperature (20–22°C) for 0.5, 4, 8, or 24 hours. Homogenized aliquots then had RNA*later* added or not. Following room temperature exposure, all aliquots were stored in the fridge (4°C) for 24 hours prior to storing in the freezer (-20°C), or stored directly in the freezer. DNA extraction, PCR amplification, then sequencing were completed on all samples. Alpha diversity (diversity, evenness, and richness), and beta diversity (community membership and structure), and relative abundances of bacterial genera were compared between treatments. Homogenization and RNA*later* minimized changes in the microbial communities over time, although minor changes in relative abundances occurred. Non-homogenized samples had more inter-sample variability and greater changes in beta diversity than homogenized samples. Storage of canine fecal samples in the fridge for 24 h prior to storage in the freezer had little effect on the fecal microbiota profile. Our findings suggest that if immediate analysis of fecal samples is not possible, samples should at least be homogenized to preserve the existing microbiota profile.

## 1 Introduction

The gastrointestinal (GI) microbiota plays an essential role in a dog’s metabolism [1], immune system function and defense against gastrointestinal pathogens [2]; its homeostasis is important for maintenance of the GI tract and overall health [3–7]. Conversely, alteration in the homeostasis of GI microbiota is associated with multiple systemic and gastrointestinal diseases [8].

The GI tract is divided into different compartments, but direct characterization of the microbial communities of those compartments is difficult and requires invasive sampling processes. Therefore, fecal samples have been consistently used as a proxy for the colonic microbiota [9]. Numerous fecal microbiota studies have been conducted in dogs, with many of them using client-owned dogs [10–12]. Fecal collection and storage practices vary among these studies, limiting the conclusions drawn and reproducibility, and preventing comparisons among different studies. The methods for sample processing, preservation, and storage temperature lack standardization in current literature and studies addressing these limitations are scarce.

In particular, methods for the allowable time for room temperature exposure, homogenization of samples, short-term storage in the fridge before freezing, and the addition of RNA*later* as a preservative require standardization. Room temperature exposure can affect the fecal microbiota profile by providing time for bacteria to undergo aerobic fermentation in the presence of oxygen [13]. Diversity and relative abundances of the feline fecal microbiota did not change with exposure to room temperature over a 4-day period [14], while noticeable alterations have been observed in fecal microbiota of horses after 6 hours of room temperature exposure [15–17]. Homogenization is often used to reduce the variability within and between fecal samples [18, 19], however, this sample preparation method may also introduce additional oxygen into a sample, potentially promoting aerobic fermentation [13]. As colder temperatures slow fermentation, short-term (i.e., 24 h) storage in the fridge prior to freezing appears to be a suitable alternative when immediate freezer storage is unavailable (e.g., in field research). However, this has primarily been investigated in human fecal samples [20, 21] and it is unclear whether those results can be extrapolated to animals. RNA*later* is a commercial reagent often used as a general DNA preservative. RNA*later* can be used to preserve the microbiota profile in fecal samples when immediate freezer storage is not possible, and has been used in canine [22] and human studies [20, 23].

While the impact of each of these variables on fecal microbiota has been studied individually, research into how they interact, particularly in dog fecal samples, is needed. Therefore, this study aimed to investigate the effects of room temperature exposure, storage in the fridge for 24 h prior to storing in the freezer, homogenization, and the addition of RNA*later* to fecal samples on the alpha- and beta diversity, and bacterial relative abundances of the canine fecal microbiota. We hypothesized that room temperature exposure would alter the fecal microbiota profile, but homogenization and RNA*later* would ameliorate those effects. Twenty-four hours of fridge storage was hypothesized to not alter the fecal microbiota profile compared to storing directly in the freezer.

## 2 Materials and methods

### 2.1 Animals and housing

Four intact female and two intact male healthy colony beagles, 1 year of age, housed at the existing dog colony from the Central Animal Facility at the University of Guelph were included in this study. Dogs were group-housed in a free-living environment and had free access to various sources of environmental enrichment such as toys and beds. For 2 h a day, 5 days a week, all dogs received human interaction with one or two familiar people. These interactions included petting, voluntary play, brushing, and general upkeep of the room. The housing space was environmentally controlled using a 12:12 light: dark regime, with lights turned on at 8:00 am and turned off at 8:00 pm. Room temperature was maintained at 20.0°C and 40–60% relative humidity. The room surfaces were sanitized and cleaned daily. All dogs were provided *ad libitum* water access and fed the same commercial diet for adult maintenance daily. In consultation with the University of Guelph Animal Care and Use Committee, an animal use protocol was deemed unnecessary for a study performing no other procedures than collecting fecal samples.

### 2.2 Experimental design

**Fig 1:**
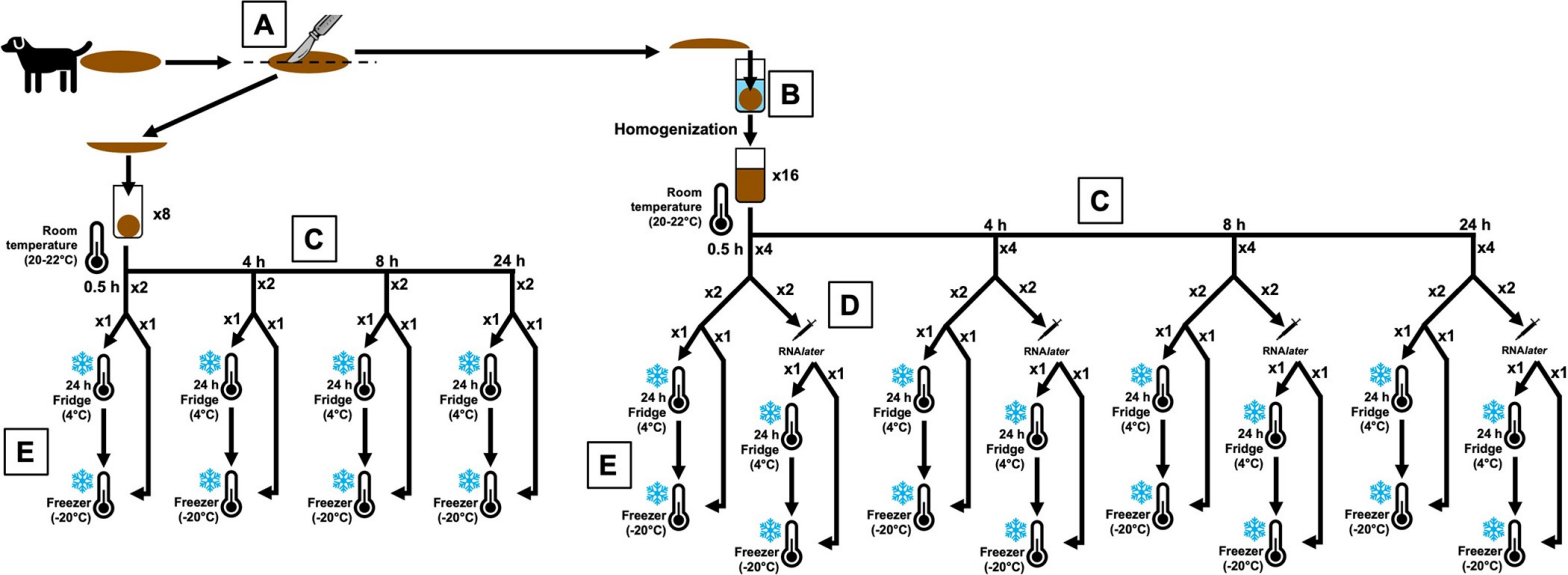
Sample collection and processing methods. From each dog, the fecal sample was cut longitudinally in half (A). One half was divided into eight non-homogenized samples, while the other half was divided into 16 homogenized aliquots (B). Four homogenized and non-homogenized samples each were stored at room temperature for 0.5, 4, 8, or 24 h (C). Of the homogenized samples at each room temperature exposure time point, two had RNAlater added (D). Samples were then frozen immediately, or stored in the fridge for 24 h before being transferred to the freezer (E).

Fecal samples were collected from each animal and processed within 30 minutes after defecation. The experimental design and sampling are summarised in Fig 1. Different sample preservation procedures were applied to the exact same fecal samples; for this purpose, each sample was divided longitudinally into equal halves (Fig 1A). The first half was equally divided into 200-mg aliquots that included the core and surface. The second half was homogenized (Fig 1B) to aid in evaluating the effects of storage temperature, room temperature exposure, and the effects of RNA*Later* on the fecal microbiota profile. From each dog, 16 aliquots of homogenized and eight aliquots of non-homogenized samples were exposed to room temperature (20–22°C) for a duration of 0.5, 4, 8, or 24 h (four homogenized and two non-homogenized samples per time point) (Fig 1C). After room temperature exposure, the effect of RNA*later* on the microbiota profile was assessed by adding RNA*later* to two of the four homogenized samples at each room temperature exposure time point at a 1:1 ratio of homogenized sample: RNA*later* (200 μL) (Fig 1D). One sample with RNA*later* was immediately frozen at –20°C and the other sample was stored at 4°C for 24 h before freezing at -20°C until further analysis was completed (Fig 1E). Similarly, for the samples without RNA*later*, one was frozen immediately at –20°C and the other was stored at 4°C for 24 h before freezing at -20°C until further analysis (Fig 1E). For the non-homogenized samples, after room temperature exposure, one of two samples at each room temperature exposure time point was immediately frozen at –20°C and the other sample was stored at 4°C for 24 h before freezing at -20°C until further analysis (Fig 1E).

Overall, six experimental groups were formed based on the combination of sample preservation procedures (when applied or not): *I)* sample homogenization (Hom or noHom), *ii*) use of the preservative RNA*Later* (Lat or noLat), and *iii*) storage temperature when preserved in the fridge for 24 hours prior to freezing (Fg) or stored directly in the freezer (Fr). Each group was named based on these conditions, for example, the group “Hom_Lat_Fg” included samples homogenized, treated with RNAlater, and stored in the fridge. In addition, each sample (and group) was analyzed at four room temperature exposure time points (i.e., 0.5, 4, 8, and 24 h), representing 24 experimental groups (n = 6), and a total of 144 fecal samples that were used for DNA extraction.

### 2.3 DNA extraction, amplification & sequencing

Using a commercial Kit (EZNA Stool DNA Kit, Omega Bio-Tek, Doraville, GA), total DNA was extracted from 200 μg of wet-weight fecal samples. Amplification of the 16S rRNA gene at the V4 region was completed with forward and reverse primers [24]. These primers were specifically designed to have overhanging adapters that allow for annealing to the sequencing adaptors used in Illumina universal index sequencing when it is added for PCR later in the process.

The amplification process of the V4 region was carried out using a reaction that consisted of 9.0 mL of molecular grade water, 12.5 mL of KAPA 2G Fas Hot Start ReadyMix 2X (KapaBiosystems), 2.5 mL template DNA, and 0.5 mL each of the reverse and forward 16S rRNA V4 primers [25]. For the PCR reaction, the samples were processed at 94°C for 10 min, followed by another 27 cycles at 94°C for 45 sec each, 60 sec at 53°C, and 90 sec at 72°C, and a final extension step of 10 min at 72°C [25]. Magnetic beads were then used to purify the PCR products (Agencourt, AMPure XP, Beckman Coulter, ON). The addition of Illumina adapters to the PCR purified 16S rRNA gene product. Using electrophoresis, the PCR products were then evaluated using agarose gel (1.5%) and purified. Once purification steps were completed, spectrometry was used for quantification of the PCR products. Samples were then submitted to the Agriculture & Food Laboratory at the University of the Guelph for sequencing with an Illumina MiSeq^c^ for 250 cycles from each end. Sequencing was carried out using 250-bp paired-end method in an Illumina MiSeq platform (Illumina Inc., USA).

### 2.4 Bioinformatic and statistical analysis

Demultiplexed sequence sets were pre-processed and analyzed using the QIIME2 pipeline v. 2021.8 [26]. Briefly, DADA2 program [27], was used via q2-dada2 for denoising and merging paired reads, including chimera removal. The resulting amplicon sequence variants (ASVs) were aligned using the MAFFT [28] via q2-alignment plugin, and the alignments were then used to construct the phylogeny following the FastTree2 method [29] as implemented in q2-phylogeny. The taxonomic classification of the ASVs was performed using the Classify-Sklearn Naive Bayes method via the q2-feature-classifier plugin. A pre-trained classifier (99%) based on 16S rRNA full-length sequences from the SILVA database v.138 was used [30].

Several phylogenetic and non-phylogenetic metrics were calculated from rarefied feature tables at the ASVs level, but Shannon entropy [31] and weighted UniFrac distance [32] were chosen to explore alpha and beta diversity, respectively. Comparisons between groups of alpha diversity metrics were performed using Kruskal–Wallis test, whereas the beta diversity simple and multiple comparisons were made using PEERMANOVA test.

To address the temporal variability within each sample, both Shannon entropy and weighted UniFrac were also analyzed using longitudinal approaches, specifically repeated measures. These analyses were carried out using the algorithms included in the q2-longitudinal plugin [33]. In essence, the comparisons between groups were centered on: *i*) Shannon entropy first differences, which refer to the differences in Shannon entropy of the same fecal sample between two time points, and *ii*) weighted UniFrac first distances, which denote differences in the weighted UniFrac distance of an individual’s microbiota composition across two distinct time points. Following this, the Kruskal-Wallis test was employed to gauge the magnitude of temporal variations among different sample groups or treatments.

Additionally, linear mixed-effects (LME) models were used to account for the influence of the predictive factors over time (i.e., DNA preservation procedures, and time) on the microbial diversity metrics, namely Shannon entropy and weighted UniFrac. The model is structured as:

$$Y_{ijklm}=\mu +A_{i}+B_{j}+C_{k}+T_{l}+{\left(A\times B\times C\times T\right)}_{ijkl}+Dog_{m}+\epsilon_{ijklm}$$

Where: *Y*_*ijklm*_ represents either Shannon entropy or weighted UniFrac, *μ* denotes the overall mean, *A*_*i*_ fixed effect of sample homogenization, *B*_*j*_ fixed effect of using the preservative RNALater, *C*_*k*_ fixed effect of storage temperature, *T*_*l*_ fixed effect of the time (hours), (A×B×C×T)_*ijkl*_ signifies the interactions among the factors, Dog_*m*_ is the random effect, accounting for individual dogs from which samples were taken and ϵ_*ijklm*_ is the random error term. The significance of the fixed effects and their interactions was assessed using F-tests. Model assumptions were verified through diagnostic plots of residuals.

Given a challenge encountered in our modeling process where the inclusion of both RNA*Later* and homogenization variables simultaneously led to model failure, we opted to conduct the analysis in two separate stages. This issue may be related to an underlying statistical complexity specific to our dataset, such as a lack of variation within certain combinations of categories. As a practical resolution, in each round of the LME, we included only one of these conflicting variables, thereby enabling successful execution of the analysis.

Longitudinal analyses based on the taxonomic profiles at the genus level were executed using the “feature-volatility” function in q2-longitudinal [33] to compare the taxonomic profile of the fecal microbiota from the different treatments.

## 3 Results

### 3.1 Sequence analysis

A total of 19,372,567 sequences were available after denoising of 16S rRNA sequence data with an average of 134,531 (SD = 25,804) sequences (merged mate-pair reads) per sample and a median frequency of 128,417 sequences. Overall, 1,470 ASVs were identified in 144 samples. Rarefaction curves showed that these sequence counts broadly covered the diversity of the microbiota in all samples.

### 3.2 Microbial diversity

**Fig 2:**
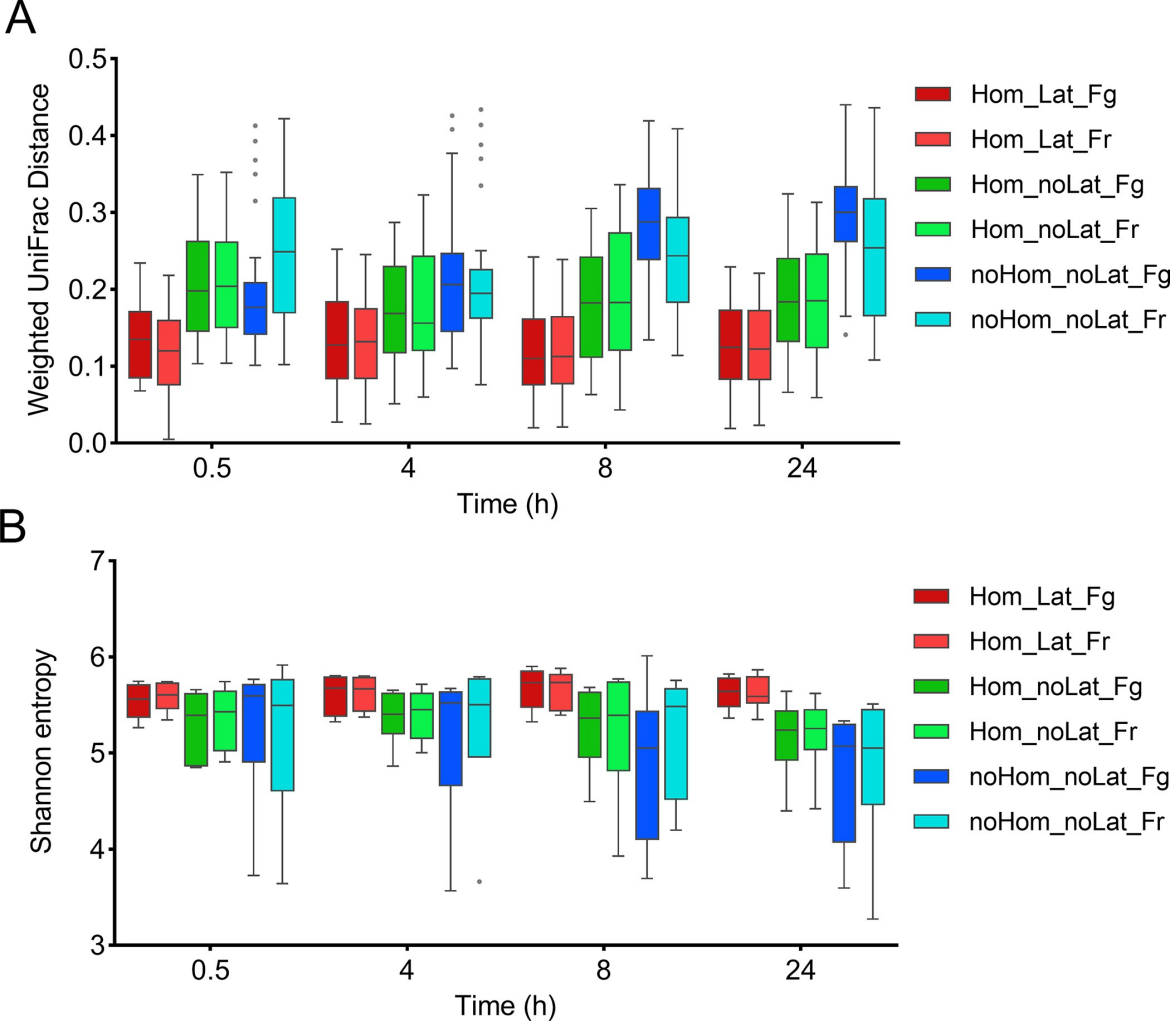
Comparison of alpha and beta diversity metrics. Pairwise comparisons of weighted UniFrac distance were performed between groups of samples subject to different feces preservation strategies **(A)**, the plot indicates the distance of each group at different time points (0.5 to 24 hours), considering homogenized samples with RNAlater added and stored in the fridge for 24 h before storing in the freezer (Hom_Lat_Fg at 0.5 h) as the reference group. Different lowercase letters in each box plot indicate significant differences between treatments (PERMANOVA test, *p* < 0.05). The Shannon entropy, which is indicative of both microbial richness and evenness, was also compared between groups, at different sampling times **(B)**. Different lowercase letters in each box plot indicate significant differences between treatments (Kruskal-Wallis test, *p* < 0.05). Hom: homogenized; Lat: RNA*later* added; Fg: fridge; Fr: freezer.

The beta diversity analysis based on weighted UniFrac revealed significant differences (*p* = 0.001) among all the groups (i.e., treatments at all the time points). Specifically, weighted UniFrac distance increased over time in the samples that were neither preserved with RNA*late*r nor homogenized (Fig 2A). Significant differences among groups were also observed on the alpha diversity metrics. The Shannon entropy differed between treatment groups (*p* = 0.01), and there was a decrease (and increased dispersion) in Shannon entropy over time in samples without RNA*later* or non-homogenized. Instead, Shannon entropy seemed to remain stable over time in samples treated with RNA*later* regardless of if they were stored in the fridge for 24 h prior to freezing or frozen immediately (Fig 2B).

A mixed linear regression analysis assessing the effect of treatments and their interactions revealed that RNA*later* had a significant effect on the Shanon entropy (*p* = 0.028), such that samples with RNA*later* had a greater Shannon entropy, whereas storage temperature and time did not have significant effects on the Shanon entropy (*p* > 0.05), and there were no significant (*p* > 0.05) interactions between any of these factors (Table 1, S1 Fig). A similar multivariate mixed linear regression analysis including homogenization instead of RNA*later* variable revealed that homogenization did not significantly affect the Shannon entropy (*p* > 0.05), and there were no interactions with room temperature exposure time or storage temperature (i.e., storing in the fridge prior to freezing versus freezing immediately) (Table 1, S1 Fig). However, the use of RNA*later*, homogenization, and the time elapsed before freezing the samples had a significant impact on microbiome composition (*p* = 0.001), as determined by PERMANOVA (Adonis) test on weighted UniFrac distance (Table 2). Storage temperature did not significantly affect beta diversity (*p* > 0.05), but a significant interaction effect (*p* < 0.05) was observed between sample homogenization and room temperature exposure time (Table 2).

**Table 1:** Mixed linear regression analysis assessing the effect of treatments (RNA*later*, storage temperature, and time for room temperature exposure prior to freezing) and their interactions on the Shannon entropy index of fecal microbiota of six healthy dogs.

**Linear mixed model 1: Addition of RNA*Later*** *	**Coef.**	**Std. Err.**	**P>|z|**
Intercept	5.592	0.176	**0.000**
RNA*later*	-0.318	0.145	**0.028**
Storage temperature	0.027	0.167	0.873
RNA*later*: Storage temperature	0.025	0.205	0.903
Time	0.002	0.009	0.798
Time: RNA*later*	-0.016	0.011	0.154
Time: Storage temperature	-0.002	0.013	0.903
Time: RNA*later*: Storage temp	0.003	0.016	0.843
**Linear mixed model 2: Homogenization** *	**Coef.**	**Std. Err.**	**P>|z|**
Intercept	5.467	0.156	**0.001**
Homogenization	-0.26	0.15	0.082
Storage temperature	0.026	0.122	0.83
Homogenization: Storage temperature	0.051	0.212	0.809
Time	-0.002	0.007	0.723
Time: Homogenization	-0.018	0.012	0.124
Time: Storage temperature	-0.001	0.01	0.907
Time: Homogenization: Storage temp	0.005	0.017	0.766

*Two models were built because the realtionship between the addition of RNA*later* to the fecal samples and the process of homogenization.

**Table 2:** Mixed linear regression analysis assessing the effect of treatments (RNA*later*, storage temperature, and time for room temperature exposure prior to freezing) and their interactions on the weighted UniFrac (beta diversity) of fecal microbiota of six healthy dogs.

Variable	SumSqs	MeanSqs	F	R^2^	Pr(>F)
RNA*later*	0.62	0.62	123.07	0.16	**0.001**
Homogenize	0.31	0.31	62.68	0.08	**0.001**
Temperature	0.00	0.00	0.66	0.00	0.566
Time	0.03	0.03	6.45	0.01	**0.002**
RNA*later*: Temperature	0.00	0.00	0.24	0.00	0.902
Homogenize: Temperature	0.01	0.01	2.13	0.00	0.097
RNA*later*: Time	0.00	0.00	0.25	0.00	0.912
Homogenize: Time	0.09	0.09	17.62	0.02	**0.001**
Temperature: Time	0.01	0.01	1.86	0.00	0.149
RNA*later*: Temperature: Time	0.00	0.00	0.85	0.00	0.449
Homogenize: Temperature: Time	0.01	0.01	1.70	0.00	0.148
Residuals	0.36	0.00	NA	0.10	NA
Total	3.74	NA	NA	1.00	NA

A longitudinal statistical approach (q2-longitudinal) was used to examine the variability of the microbial diversity metrics in each fecal sample from 0.5 to 24 h. Specifically, for this analysis, the alpha diversity metric was represented by the "Shannon entropy first difference" and the beta diversity metric by the "weighted UniFrac first distance". These metrics capture the changes in Shannon entropy and weighted UniFrac over time for each sample.

**Fig 3:**
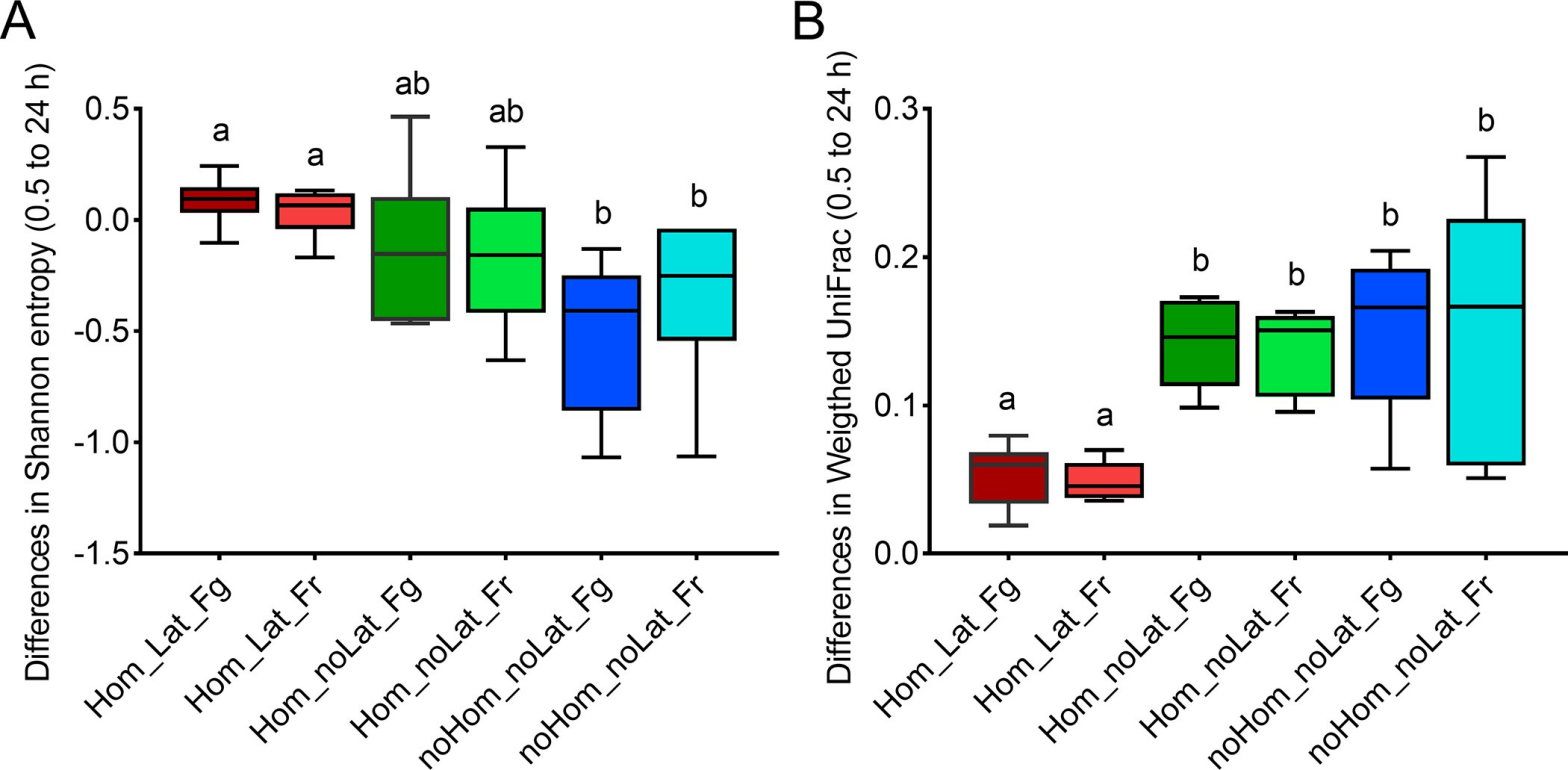
Longitudinal analysis of alpha and beta diversity in different treatment groups. The temporal variations in Shannon entropy (from 0.5 to 24 h) were compared between groups **(A)**. Pairwise comparisons were also performed on the differences of weighted UniFrac distance between 0.5 to 24 h **(B)**. Different lowercase letters in each box plot indicate significant differences between treatments (Kruskal-Wallis test, *p* < 0.05). Hom: homogenized; Lat: RNA*later* added; Fg: fridge; Fr: freezer.

Homogenization of the feces and the addition of RNA*later* preserved the fecal microbiota, as demonstrated by the reduction in the Shannon entropy index only in homogenized samples without RNA*later* (S2A Fig). Shannon entropy first difference (i.e., differences between 0.5 and 24 h) analysis revealed that alpha diversity decreased (*p* = 0.004) over time in samples that were neither homogenized nor treated with RNA*later* (S2A Fig). Although there was no statistically significant difference between homogenized samples with and without RNA*later*, a decrease in Shannon entropy first difference was observed in homogenized samples without RNA*later* (Fig 3A). The random forest analyses also showed that the weighted UniFrac distances remained stable within the homogenized samples with added RNA*later* regardless of the storage temperature. Those changes in beta diversity were evident even in samples processed at 4 h (S2B Fig). The weighted UniFrac first distance increased (*p* = 0.002) in samples that were not treated with RNA*later* (Fig 3B). Differences in Shannon entropy first difference or weighted UniFrac first distance were not identified between samples stored in the fridge for 24 h prior to freezing and sample frozen immediately (Fig 3).

### 3.3 Taxonomic composition

A total of 136 taxa were detected at the genera level, most of them from the phyla Bacillota (former Firmicutes) (74%), followed by Pseudomonadota (Proteobacteria) (11%) and Bacteroidota (10%). The most abundant bacterial order were Erysipelotrichales, Oscillospirales, and Peptostreptococcales-Tissierellales. Taxonomic analysis at the genus level revealed that different sample preservation techniques changed the proportional abundance of several taxa (S3 Fig), with these changes more evident at 24 h in samples that were neither homogenized nor treated with RNAlater (S4 Fig).

**Fig 4:**
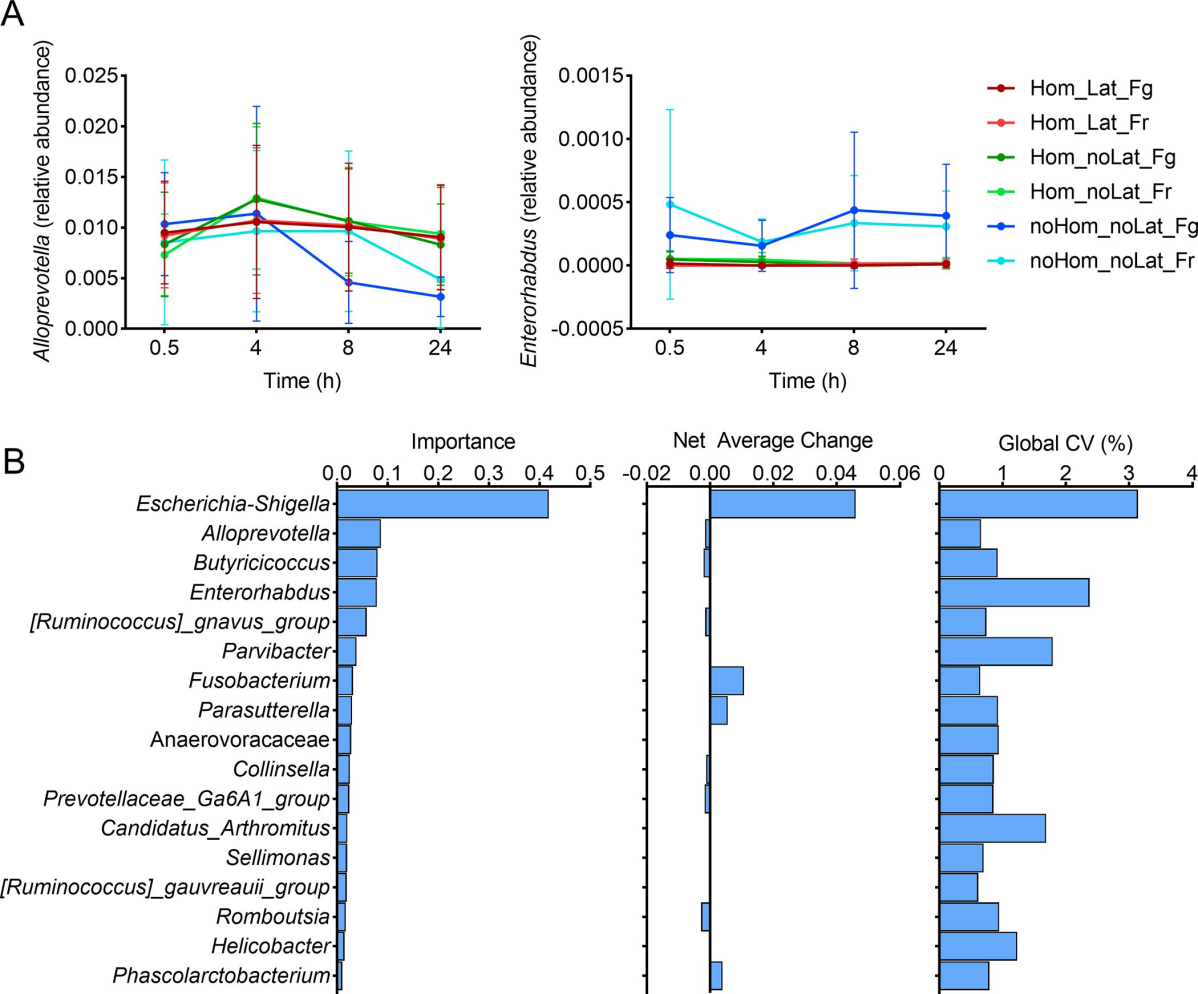
Longitudinal “feature-volatility” analysis of bacterial genera abundance. Relative abundances of *Alloprevotella* and *Enterorhabdus* over time are shown for each group of samples (i.e., from each feces preservation treatment). The mean and standard deviation are indicated in each group **(A)**. The 17 taxa detected with the temporal variation signal are shown, ranked in order of importance, with other descriptive statistics (net average change, coefficient of variation) included **(B)**. Hom: homogenized; Lat: RNA*later* added; Fg: fridge; Fr: freezer.

To identify bacterial genera affected by the sample preservation procedures, a longitudinal machine learning analysis (random forest analysis) was conducted. Overall, 17 bacterial genera had high importance explaining the variability in the taxonomic composition of the microbiota across all samples over time, which were particularly evident in non-homogenized samples or samples without RNA*later* (Fig 4). Of interest, *Escherichia-shigella* had the highest importance explaining microbial changes overtime. These analyses employed machine learning regressors (random forests) to identify "important" features (bacterial genera) that change over time, indicating a temporal relationship. Importantly, feature importance does not imply statistical significance.

## 4. Discussion

This study investigated the effects of homogenization, the addition of RNA*later*, room temperature exposure, and storage temperature on the canine fecal microbiota. The main findings were that homogenization of fecal samples and addition of RNA*later* prevented changes in the microbial communities present in feces of healthy dogs, although minor changes in relative abundances of specific taxa occurred. Non-homogenized samples also had more inter-sample variability and greater changes in beta diversity than homogenized samples. Finally, the present study showed that storage of fecal samples from colony beagles in the fridge at 4°C for 24 h prior to storage in the freezer at -20°C had little effect on the fecal microbiota profile.

Homogenization of fecal samples prior to storing at 4°C for 24 h before freezing reduced the intra-individual variability of the fecal microbiota. Non-homogenized samples had major changes in alpha- and beta diversity. Gorzelak et al. [19] observed similar findings in humans as they showed that homogenization of human fecal samples (albeit in liquid nitrogen) prior to subsampling reduced the variability of bacterial taxa detected by qPCR. Similarly, Hsieh et al.[18] found that homogenization decreased intra-individual variation in alpha diversity of human fecal samples, and had little effect on the relative abundance of dominant taxa. However, the relative abundances of some specific genera were impacted (i.e., *Faecalibacterium*, *Streptococcus*) by homogenization in the study by Hsieh et al. [18]. The authors proposed two hypotheses for the effect of homogenization on some taxa: first, some taxa are sensitive to lysis and DNA degradation, resulting in reduction of their abundance after homogenization; or second, homogenization can dislodge bacteria from macromolecules or aggregates, making these taxa available during DNA extraction and leading to a higher detection rate of dislodged bacteria [18]. The latter hypothesis can be supported by the results of the present study as homogenization of fresh fecal samples before storage at 4°C or -20°C reduced the intra-sample variability. However, homogenization of samples in field settings (e.g., from client-owned animals) is not always possible, therefore, future studies should determine whether the effect of homogenizing a sample or not is so large that it would conceal the effects of an intervention.

RNA*later* reduced sample variability but resulted in no alteration of the alpha- and beta diversity of the fecal samples from healthy dogs. Contrary, several previous studies showed an alteration of the alpha diversity indices of the human fecal microbiota when RNA*later* was added to the samples [20, 22]. A study comparing several fecal microbiota preservatives found that RNA*later* alters beta diversity at a higher magnitude than 70% ethanol and 50:50 glycerol:PBS [22]. The latter results may differ from the present study because the authors collected one fecal sample from one dog (compared to the present study’s, which comprised samples from six dogs), and a larger sample size can introduce a greater inter-individual variation. Effects of RNA*later* on the relative abundances of various phyla in the fecal microbiota of children [23] and genera in the canine fecal microbiota [22] were reported. Although not observed in this study, the results of several studies [20, 22, 23] have raised the question of whether, in acting to preserve a sample’s DNA, RNA*later* causes alterations to the relative abundances of the fecal microbiota profile. Liang et al. [23] theorized that changes to bacterial relative abundances arise because RNA*later* reduces DNA purity and decreases DNA yield during DNA extraction, leading to a reduction in highly abundant bacteria and potentially loss of bacteria with a normally low abundance. Although RNA*later* showed clear ability to reduce the changes in alpha and beta diversity from 0.5 to 24 h post-collection in the present study, the ability for RNA*later* itself to alter the microbiota profile should be considered in future research using RNA*later* during storage. While the present study showed that the use of a DNA preservative could be a reasonable approach for preservation of canine fecal samples, our results suggest that if a DNA preservative is not available, homogenization alone will help to preserve the DNA and produce more precise data (i.e., lower variability).

The present study observed no effect of storing in the fridge at 4°C for 24 h prior to freezing at -20°C on the canine fecal microbiota profile. These findings are consistent with previous studies on human fecal samples showing no effect of storing in the fridge for 24 h to 72 h prior to freezing at -80°C on the fecal microbiota profile [20, 21, 34]. By contrast, a study using fecal samples from healthy dogs showed that long-term (7 to 56 days) storage at 4°C significantly affected beta diversity and relative abundances of the main taxa compared to samples stored immediately at -80°C [22]. While we did not compare between fecal samples stored at -20°C and -80°C, others suggest there is no difference [21, 35]. Our study showed that short-term (< 24 h) storage of canine fecal samples at 4°C prior to microbiota analysis is acceptable. These results are of special importance in circumstances when immediate storage in the freezer is not possible, such as field research or collection from client-owned dogs in home settings.

Although no effect of time was observed on alpha and beta diversity of the canine fecal microbiota across 24 h of room temperature exposure in the present study, the relative abundances of some bacterial genera changed over time, most notably *Escherichia-shigella*. These findings are similar to those previously reported in humans and cats. The microbiota profile of feces from children were not altered in homogenized samples stored at ambient temperature for a 32h period regardless of any DNA preservative added to the sample [36]. Additionally, fecal samples from humans stored at room temperature for 14 days had minimal effect on relative abundances of the main taxa [37]. Similar findings were reported in fecal samples from healthy cats exposed to room temperature for a 96-h period [14]. By contrast, a study using fecal samples from healthy horses showed that the relative abundance of the main phyla and genera are affected by 6 h of room temperature exposure [15]. Another study on humans observed no effect of 24 h of room temperature exposure on the stability of the fecal microbial community of healthy adults, however, fecal samples from adults with irritable bowel syndrome were more affected [38]; therefore, it is unclear whether our results would translate to dogs with gastrointestinal conditions. In the present study, the relative abundance of *Escherichia-Shigella* increased in non-homogenized samples when feces were stored at room temperature. This is of importance because this taxon is frequently reported in dogs with gastrointestinal dysbiosis, and it is associated with local and systemic disease [39–41]. Thus, researchers should establish sample collection and storage protocols aiming to preserve the integrity of the fecal microbiota when designing studies investigating changes in microbial communities in diseased animals compared to healthy ones. These protocols are of major importance when using client-owned animals.

Although this study’s convenience sample of six dogs was greater than previous studies, it is possible that different effects would have been observed, including more noticeable differences between treatments, such as homogenized versus non-homogenized and room temperature exposure duration, with a greater sample size. The animals included in the present study are also research dogs living in the same environment and consuming the same diet, limiting comparability to the overall population. Additionally, the present study only assessed four time points (0.5, 4, 8, and 24 h post-collection). Using smaller intervals between time points (e.g., 0.5, 2, 4, 6, 8 h, etc.) or a longer period such as 96 h [14] could have provided more detailed information regarding variations in the fecal microbiota profile over time with room temperature exposure. The present study also only assessed one type of preservative (RNA*later*), limiting applicability of the results to other types of preservatives such as 70% ethanol and 50:50 glycerol:PBS, which have also been found to impact the fecal microbiota profile over time [22]. As well, though we observed that some storage conditions led to more variation, the clinical utility is unknown until future studies explore whether the differences we observed are masked when adding a relevant target population (e.g., a nutritional intervention, antibiotics, or chronic enteropathies). There is also potential for analytical variability in the sequencing pipeline. While we applied rigorous efforts to minimize technical biases, intra- and inter-assay variation, end-of-run variation, and other sources of variability within the sequencing process could impact microbial stability and were unfortunately beyond the scope of the present study. Future research can explore the gaps left by these limitations to build on the present study’s findings and provide further clarity to optimize methods used in the study of the canine fecal microbiota.

## 5. Conclusion

The present study’s findings suggest that if fecal samples are not to be analyzed immediately, they should at least be homogenized to preserve the existing microbiota profile. Further investigation is required to determine if the ability for RNA*later* to alter the microbiota profile can be detrimental, and if there are any deleterious effects on DNA purity [23]. Our findings will aid in protocol development for future studies, particularly in field research and research on client-owned companion animals; however, more research is needed to further standardize sample collection and storage protocols in microbiota research.

## Supporting information

S1 Fig Regression scatterplots of the Shanon entropy as determined by the predictor variables: RNA*later* **(A)**, Homegenizacion of the feces (**B)**, and storage temperature **(C)**. Hom: homogenized; Lat: RNA*later* added; Fg: fridge; Fr: freezer.
(TIF)

S2 Fig Feature volatility plots of alpha **(A)** and beta **(B)** diversity metrics from each sample group over time (between 0.5 and 24 h). In each group, the mean line and the standard error bar are included. Hom: homogenized; Lat: RNA*later* added; Fg: fridge; Fr: freezer.
(TIF)

S3 Fig Taxonomic profiling of the fecal microbiota of healthy dogs discovered at various timepoints, resulting from various sample preservation techniques. The identity and abundance of taxa are displayed at the genus level. Each bar represents the average of six samples per treatment. Hom: homogenized; Lat: RNA*later* added; Fg: fridge; Fr: freezer.
(TIF)

S4 Fig Dendrogram heatmap of the taxonomic profile at genera level of the whole data set. The feature counts were normalized using center log ratio transformation. Only top 50 taxa were included, based on their hierarchical clustering. Hom: homogenized; Lat: RNA*later* added; Fg: fridge; Fr: freezer.
(TIF)
