# Supplementary figures and images for: Impact of fecal sample preservation and handling techniques on the canine fecal microbiota profile
Source: PLoS One. 2024 Jan 29;19(1):e0292731. doi: 10.1371/journal.pone.0292731 (PMC10824447; doi:10.1371/journal.pone.0292731)

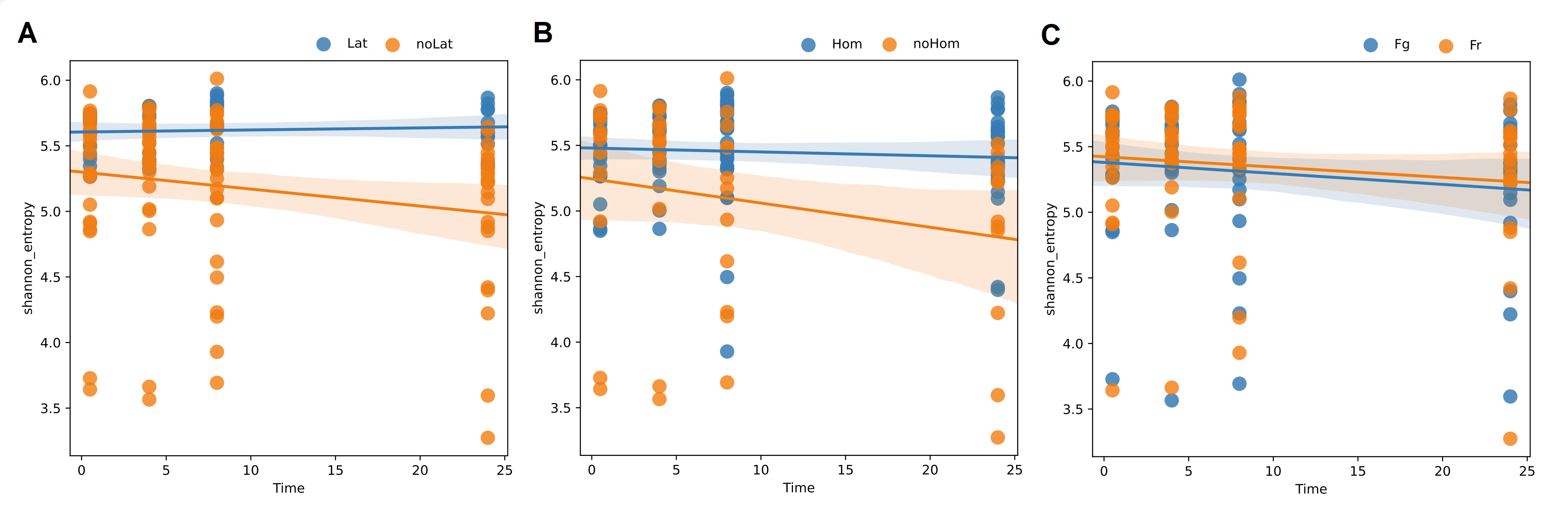

Supplement: S1 Fig — Regression scatterplots of the Shanon entropy as determined by the predictor variables: RNAlater (A), Homegenizacion of the feces (B), and storage temperature (C). Hom: homogenized; Lat: RNAlater added; Fg: fridge; Fr: freezer. (TIF) [file pone.0292731.s001.tif]

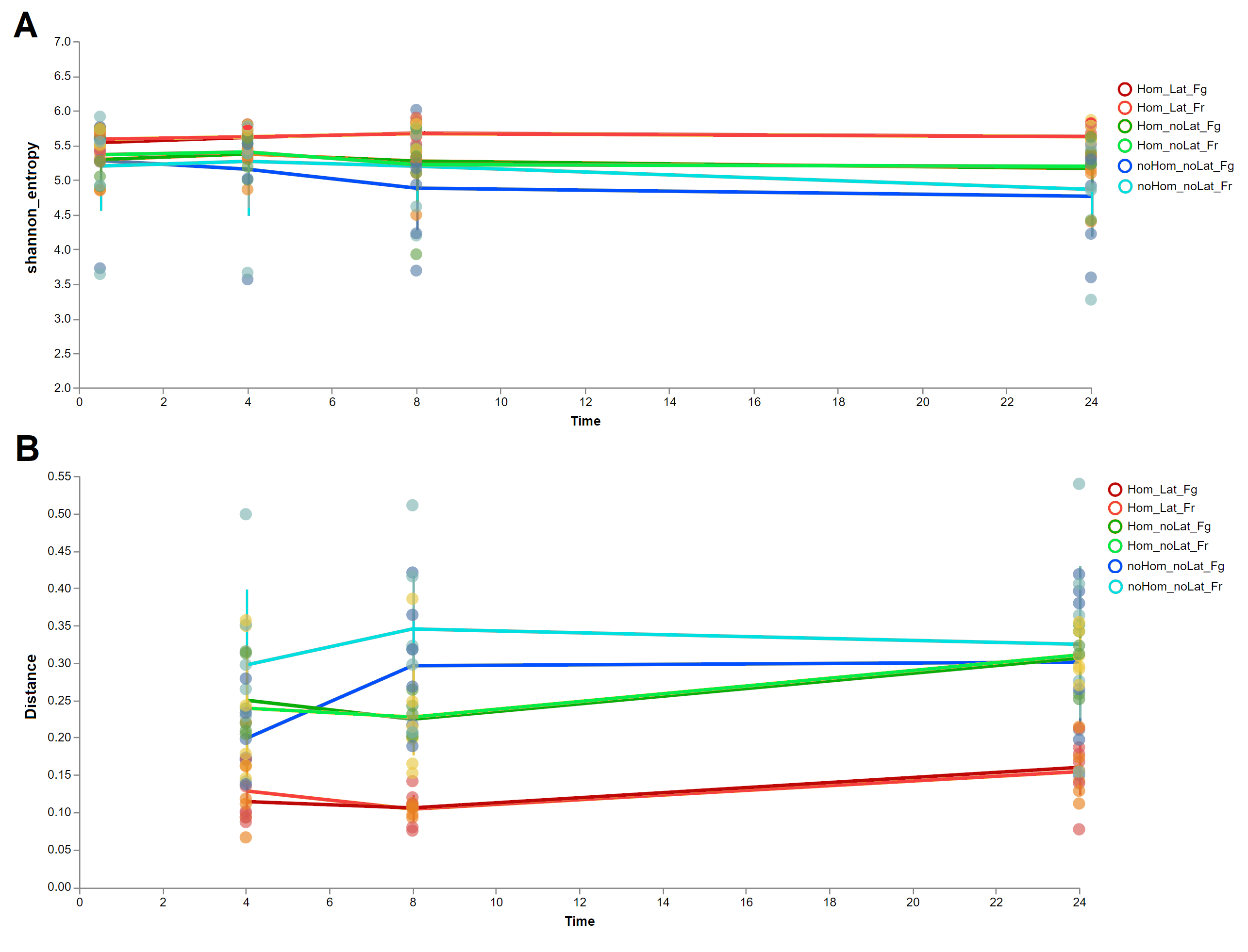

Supplement: S2 Fig — Feature volatility plots of alpha (A) and beta (B) diversity metrics from each sample group over time (between 0.5 and 24 h). In each group, the mean line and the standard error bar are included. Hom: homogenized; Lat: RNAlater added; Fg: fridge; Fr: freezer. (TIF) [file pone.0292731.s002.tif]

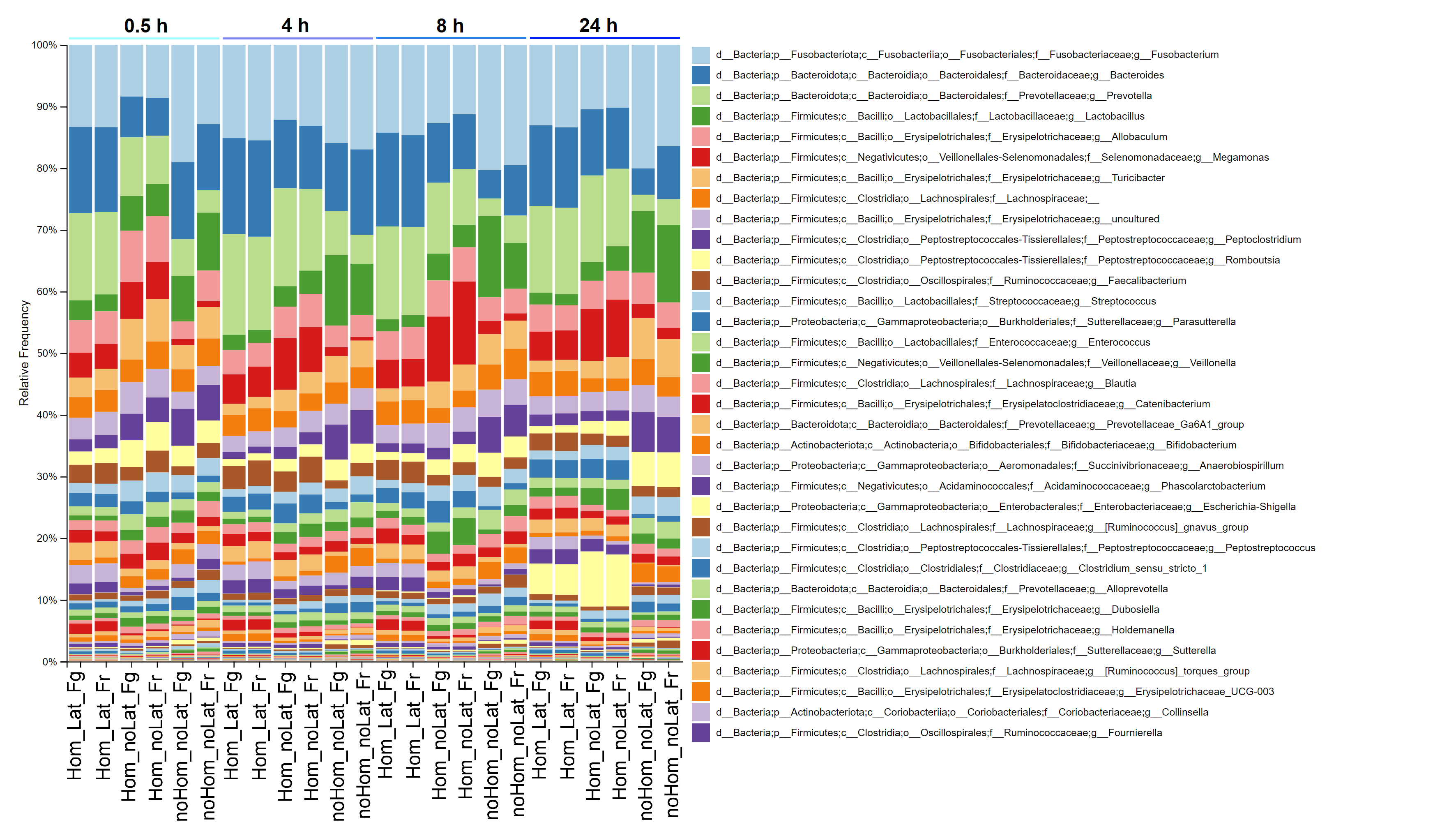

Supplement: S3 Fig — The identity and abundance of taxa are displayed at the genus level. Each bar represents the average of six samples per treatment. Hom: homogenized; Lat: RNAlater added; Fg: fridge; Fr: freezer. (TIF) [file pone.0292731.s003.tif]

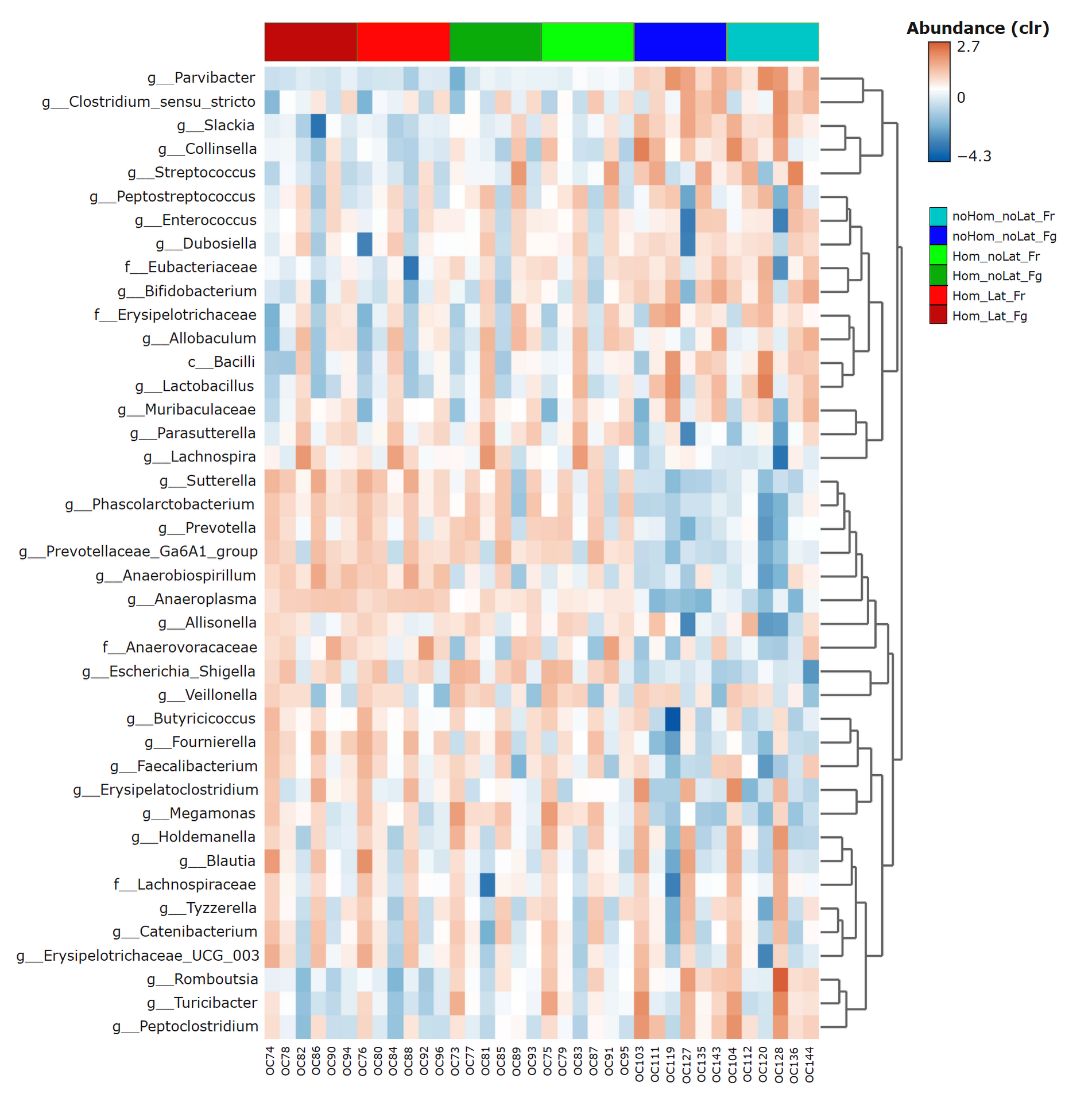

Supplement: S4 Fig — The feature counts were normalized using center log ratio transformation. Only top 50 taxa were included, based on their hierarchical clustering. Hom: homogenized; Lat: RNAlater added; Fg: fridge; Fr: freezer. (TIF) [file pone.0292731.s004.tif]
